# Optimization of a magnetic capture RT-LAMP assay for fast and real-time detection of potato virus Y and differentiation of N and O serotypes

**DOI:** 10.1007/s00705-017-3635-3

**Published:** 2017-11-08

**Authors:** Krzysztof Treder, Joanna Chołuj, Bogumiła Zacharzewska, Lavanya Babujee, Mateusz Mielczarek, Adam Burzyński, Aurélie M. Rakotondrafara

**Affiliations:** 1 Laboratory of Molecular Diagnostic and Biochemistry, Bonin Research Center, Plant Breeding and Acclimatization Institute-National Research Institute, 76-009 Bonin, Poland; 2 Department of Plant Pathology, University of Wisconsin-Madison, 1630 Linden Drive, Madison, WI 53706 USA; 3 NOVAZYM POLSKA s.c., Poznań Science and Technology Park, Rubież 46H Street, 61-612 Poznan, Poland

## Abstract

Potato virus Y (PVY) infection has been a global challenge for potato production and the leading cause of downgrading and rejection of seed crops for certification. Accurate and timely diagnosis is a key for effective disease control. Here, we have optimized a reverse transcription loop-mediated amplification (RT-LAMP) assay to differentiate the PVY O and N serotypes. The RT-LAMP assay is based on isothermal autocyclic strand displacement during DNA synthesis. The high specificity of this method relies heavily on the primer sets designed for the amplification of the targeted regions. We designed specific primer sets targeting a region within the coat protein gene that contains nucleotide signatures typical for O and N coat protein types, and these primers differ in their annealing temperature. Combining this assay with total RNA extraction by magnetic capture, we have established a highly sensitive, simplified and shortened RT-LAMP procedure as an alternative to conventional nucleic acid assays for diagnosis. This optimized procedure for virus detection may be used as a preliminary test for identifying the viral serotype prior to investing time and effort in multiplex RT-PCR tests when a specific strain is needed.

## Introduction

Potato virus Y (PVY) is the type member of the genus *Potyvirus*, family *Potyviridae* [9]. It has recently been listed among the 10 most important plant viruses in the world [12] and recognized as the major virus infecting potato [8]. A major challenge in the control of PVY infection is the continual emergence of new recombinants derived mostly from the ordinary PVY^O^ strain and the necrotic PVY^N^ strain [2, 5, 7]. At least nine PVY strains are currently known and differ at the biological, serological, and molecular levels but can be grouped into two serotypes: the O serotype, which includes PVY^O^, PVY^N:O^, PVY^N-Wi^, and PVY^C^, and the N serotype, which includes PVY^N^, PVY^E^, PVY^Z^, PVY^NA-N^ and PVY^NE11^ [8]. Due to their economic importance [2, 8], the identification of PVY isolates by strain, or at least by serotype, is needed. Many procedures for the detection of PVY in potato leaves and tubers have been reported [3, 6, 13–16]. Due to their easy adaptation to automation, their high specificity, and their ability to detect viral strains in a single reaction, real-time-RT-PCR-based assays appear to be ideal successors of the double sandwich enzyme-linked immunosorbent assay (DAS-ELISA) that is routinely used in the grow-out test of dormant tubers for the diagnosis of potato viruses for seed certification [3]. However, such methods have not yet been adapted for routine diagnostic schemes.

Here, we describe a streamlined magnetic capture version of the reverse transcription loop-mediated amplification (MC-RT-LAMP) assay that we optimized for fast detection and discrimination of PVY by their serotypes. To contrast this assay with previously reported RT-LAMP variants for the detection of PVY [1, 10, 11], we designed a primer set that amplified a conserved region within the coat protein gene that contains two single and two double nucleotide signatures typical for O and N coat protein types, which differ in their annealing temperatures in the RT-LAMP assay depending on the serotype of the virus tested. This allowed us to rapidly distinguish the PVY O and PVY N types in one reaction. In addition, the optimization of the RNA isolation step by magnetic capture, both from potato leaves and tubers, shortened the procedure while retaining its high sensitivity. The present study offers a fast alternative to conventional nucleic-acid-based assays and to the routinely used DAS-ELISA for grow-out tests, which can be time consuming, for the direct identification of PVY isolates.

## Materials and methods

### Source of plants and viruses

Potato tubers (cultivars Liwia, Nysa and Wilga) infected with PVY isolates 12/94, N Nysa, Wi and LW were obtained from the Młochów Research Center of the Plant Breeding and Acclimatization Institute – National Research Institute. The PVY isolates PV-0403, PV-0410, PV-0348, PV-0345, and PV-0343 were purchased from the plant virus collection maintained by the Leibniz Institute DSMZ - German Collection of Microorganisms and Cell Cultures. Leaf samples infected with PVY isolates Bonin 1-3 were collected in local experimental fields. Strain characteristics and GenBank accession numbers (where available) are shown in Table 1.

**Table 1:** Description of the PVY isolates used in the study

Isolate	Strain	Serotype	Accession number	Country	Isolate source
12/94	PVY^NTN^	N	AJ889866	Poland	Młochów
Wi	PVY^N-Wi^	O	EF558545	Poland	Młochów
N Nysa	PVY^N^	N	FJ666337	Poland	Młochów
LW	PVY^O^	O	AJ890349	Poland	Młochów
PV-0403	PVY^NTN^	N	–	Hungary	DSMZ
PV-0410	PVY^NTN^	N	–	Germany	DSMZ
PV-0348/CH-605	PVY^N^	N	X97895	Switzerland	DSMZ
PV-0327	PVY^N^	N	–	France	DSMZ
PV-0345	PVY^O^	O	–	Spain	DSMZ
PV-0343	PVY^O^	O	–	Germany	DSMZ
Bonin 1	PVY^O^	O	–	Poland	Bonin
Bonin 2	PVY^N-Wi^	O	–	Poland	Bonin
Bonin 3	PVY^NTN^	N	–	Poland	Bonin
MN-2	PVY^NTN^	N		USA	Madison
Tu 660	PVY^NA-NTN^	N	AY166866	USA	Madison
NTN40 D8A	PVY^NTN^	N	-	USA	Madison
Pito	-	N	-	USA	Madison
PVY:O	-	O	-	USA	Madison
CR-1	PVY^N:O^	O		USA	Madison
ID-1	PVY^N:O^	O	DQ157178	USA	Madison
PB22	PVY^N:O^	O	-	USA	Madison

DSMZ, the Leibniz Institute DSMZ (German Collection of Microorganisms and Cell Cultures)

Potato tubers of cultivars Osa, Giewont, Leona, and Pungo infected with potato leafroll virus (PLRV), potato virus M (PVM), potato virus S (PVS), and potato virus X (PVX), respectively, and virus-free tubers of cultivar Sagitta were obtained from the Laboratory of Genetic Resources and In Vitro Cultures of the Department of Potato Protection and Seed Science in Bonin, Plant Breeding and Acclimatization Institute National Research Institute.

Unless indicated otherwise, PVY isolate 12/94 (PVY^NTN^) was used in all experiments.

### RNA isolation

Total RNA was extracted using different methods as indicated. The initial sap was extracted from approximately 100-200 mg of potato leaf or tuber tissue.

#### Silica capture procedure

Total nucleic acid was purified using the silica capture procedure as described by Zacharzewska et al. [16]. Nine hundred microliters of L6 buffer (5.25 M guanidinium thiocyanate, 50 mM Tris-HCl, pH 6.4, 20 mM EDTA, and 1.3% Triton X-100) was added to 50 µl of silica beads and mixed with 100 µl of undiluted plant sap. After a 10-min incubation at room temperature with gentle shaking, the samples were centrifuged at 12,000*g*. The pellet was next washed twice with 1 ml of L2 buffer (5.25 M guanidinium thiocyanate, 50 mM Tris-HCl, pH 6.4), twice with ethanol, and once with acetone. The final pellet was dried, resuspended in 50 µl of RNase-free water, and incubated for 10 min at 56 °C. The samples were next quickly centrifuged, and the supernatants containing total nucleic acids were collected.

#### Manufacturer’s magnetic capture procedure

Total RNA was purified from 100 µl of undiluted sap using a Novabeads Total RNA Purification Kit (Novazym Polska s. c.) according to the manufacturer’s protocol.

#### Modified magnetic capture procedure

All steps and buffers were as described as above for the silica capture method except that the silica beads were replaced with 25 µl of magnetic particle suspensions from the Novabeads Total RNA Purification Kit (Novazym Polska s. c.). All centrifugation steps were replaced by the use of a magnetic stand.

#### Shortened magnetic capture procedure

This is a shortened version of the modified magnetic capture procedure. One hundred microliters of undiluted sap was added to 25 µl of Novabeads magnetic particles (Novazym Polska s. c.), which were resuspended in L6 buffer. The samples were incubated at room temperature with gentle shaking for 10 min. Next, the magnetic particles were captured on a magnetic stand, and the supernatants were removed. The beads were washed with L2 buffer and then with 70% ethanol. The beads were finally resuspended in 50 µl of RNase-free water and incubated for 5 min at 56 °C prior to collection of the supernatants.

For RT-LAMP and real-time RT-PCR, total nucleic acid preparations were made without prior DNAse treatment. Each RNA isolation was conducted with at least two replicates and was repeated independently at least three times.

### RT-LAMP assay

**Fig. 1:**
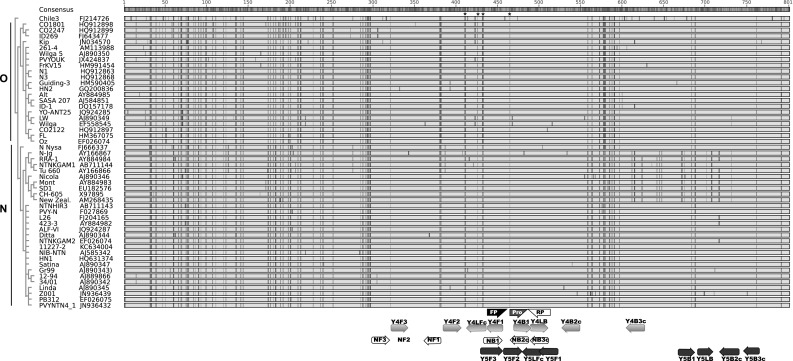
Alignment of PVY coat protein sequences with the positioning of the primer sets. The open reading frames of the coat protein (cp) genes of 50 PVY isolates representing O and N types were aligned, and a phylogenetic tree was built (shown in blue) using default setting in Genious Pro 5.0.4 software (Biomatters Ltd.). The annotated base positions 1-801 corresponds to bp 8480-9280 in the genome of PVY isolate N Nysa (FJ666337). The isolate names are followed by their corresponding accession numbers. Non-potato-infecting isolate Chile 3 was used as an out-group. The consensus sequence represents bases matching at least 75% of the aligned sequences. Only disagreements to the consensus are shown as color-coded. Nucleotides in the consensus sequence are color-coded as follows: red, A; yellow, G; blue, C; green, U; gray, ambiguous (R, Y, M). The positions of the RT-LAMP primer sets (Y4, Y5 and N), the real-time RT-PCR primers (FP, RP), and the probe (Pro) are indicated under the alignment. F1 corresponds to the sequence complementary to the F1c region of the FIP primer. B2c corresponds to the sequence complementary to the B2 region of the BIP primer. LFc and B3c correspond to sequences complementary to the LF and B3 primers. The direction of the arrows indicates the 5′-3′ orientation of the sequences shown in Table 2. The regions amplified by FIP and BIP primers of the N, Y5 and Y4 sets corresponded to nt 8808-8965, nt 8938-9217 and nt 8865-9027, respectively. Asterisks indicate the four nucleotide signatures specific to the O and N coat protein types, at nt 8890 and nt 8891 (GR for O type and AR for N type), nt 8905 (G for O type and A for N type), nt 8911 and nt 8912 (CU for O type and AC for N type) and nt 9035 (C for O type and A for N type). R represents either A or G

Different primer sets were tested in this study, including the N set developed by Nie [10], the Y5 set developed by Przewodowska et al. [11], and a new Y4 primer set that we designed using LAMP Designer software (Premier Biosoft) as described by Przewodowska et al. [11] (Table 2). All primer sets consisted of four (N set) or six (Y4 and Y5 sets) primers and targeted different regions of the PVY coat protein gene (Fig. 1). The RT-LAMP reaction mixture (10 μl) contained 0.375 µM each of outer primer (F3, B3), 1.5 µM each of inner primer (FIP, BIP), 0.75 µM each of loop primer (LF, LB), 1X Isothermal Master Mix containing proprietary fluorescent dye (Novazym Polska sc.), 0.25 U of AMV reverse transcriptase (Novazym Polska s. c.), and 100 pg of total RNA. The amplification was performed either with a Genie II Ultra rapid amplification instrument (OptiGene Ltd.) or with a CFX96 Touch™ Real-Time PCR Detection System (Bio-Rad Ltd). With the OptiGene instrument, the assay was conducted at 65 °C for 30 min. The annealing temperature (T_a_) of the amplified products was analyzed in a slow annealing step (0.05 °C/s) for 5 min, starting at 95 °C and ending at 80 °C, with monitoring of the fluorescence. The thermal profile with the Bio-Rad instrument included 60 cycles of 30 s at 65 °C. The amplification was followed by the analysis of the melting temperature (65 °C to 98 °C, 0.5 °C/s).

**Table 2:** The primer sets used for RT-LAMP, RT-PCR and real-time RT-PCR. The F1c and B1c regions of the FIP and BIP primers are indicated by lower-case letters, and the F2 and B2 regions of the FIP and BIP primers are shown as upper-case letters

Name	Position^a^ (nt)	Sequence
		**RT-LAMP Y4 set**
Y4F3^b^	8802-8820	TGC CAA CTG TGA TGA ATG G
Y4B3^b^	9105-9085	GTT CGT GAT GTG ACC TCA TAA
Y4FIP	F1c 8934-8917 F2 8865-8884	gca ttc tca acg att ggt ACG GAG TTT GGG TTA TGA TG
Y4BIP	B1c 8950-8968 B2 9027-9008	gca aat cat ggc aca ttt c CG TGG CAT ATA TGG TTC CTT
Y4LF	8914-8893	CAA TGG GTA TTC GAC TTG TTC A
Y4LB	8969-8987	TCA GAT GTT GCA GAA GCG T
		**RT-LAMP Y5 set, Przewodowska et al. [** 11 **]**
Y5F3	8910-8931	CGT TGA AAC CAA TCG TTG AGA A
Y5B3	9241-9224	GAC ATC CTC GGT GGT GTG
Y5FIP	F1c 9001-8981 F2 8938-8958	gcg cat ttc tat ata cgc ttc ACC AAC CCT TAG GCA AAT CAT
Y5BIP	B1c 9145-9162 B2 9217-9169	cgc agc att gaa atc agc TGT GTT CTC CTC TTG TGT ACT G
Y5LF	8980-8960	TGC AAC ATC TGA GAA ATG TGC
Y5LB	9168-9184	CTC GAC TTT TCG GGT TGG A
		**RT-LAMP N set, Nie [** 10 **]**
NF3	8779-8799	ATA CGA CAT AGG AGA AAC TGA
NB3	8987-8968	ACG CTT CTG CAA CAT CTG AG
NFIP	F1c 8859-8841 F2 8808-8829	gtt tgg cga ggt tcc att ttc TGT GAT GAA TGG GCT TAT GGT
NBIP	B1c 8913-8933 B2 8965-8945	tga aac caa tcg ttg aga atg ATG TGC CAT GAT TTG CCT AAG
		**TM real time RT-PCR set, Singh et al. [** 13 **]**
Y1 FP	8918-8939	CCA ATC GTT GAG AAT GCA AAA C
Y1 RP	8991-8967	FAM-ATA TAC GCT TCT GCA ACA TCT GAG A-BHQ1
Y1 probe	8946-8965	TTA GGC AAA TCA TGG CAC AT

^a^ The nucleotide position corresponds to the genome of PVY isolate N Nysa with accession number FJ666337

^b^ Primers used as forward (Y4F3) and reverse (Y4B3) primers in conventional RT-PCR, in SG-based real-time RT-PCR, and in TM-based real-time RT-PCR with probe Y1

### Strain identification by multiplex RT-PCR

Total RNA was isolated according to Zacharzewska et al. [16] from tobacco leaves (cv. Samsun) infected with PVY isolates Bonin 1-3, PVY^O^ (PV-0345), PVY^N-Wi^ (EF558545), PVY^N^ (PV-0348), and PVY^NTN^ (PV-0403). Reverse transcription reactions were carried out using a Reverse Super Verte KIT with random primers (Novazym Poland), following the manufacturer’s protocol. The resulting cDNA was next amplified by multiplex PCR according to Chikh-Ali et al. [4] with some modifications. The PCR mixture (10 µl) contained: 1-3 µl of cDNA; 0.2 U of uracil DNA glycosylase (UNG) (Bioline); 0.4 mM dNTPs (including dU instead of dT); 2 mM MgCl_2_; 0.2 mM primers n156, o514, n787, o2172, o2700; 0.1 mM primers n2258, n2650c; 0.6 mM primers n7577, SeroN, Y03-8648; 1 µl of 10-fold-concentrated polymerase buffer, and 0.6 U of GoTaq HotStart Polymerase (Promega). The temperature profile followed was as follows: UNG clean up at 40 °C for 2 min and initial denaturation at 94 °C for 1 min; 10 cycles at 94 °C for 30 s, 64 °C for 30 s, and 72 °C for 30 s; 10 cycles at 94 °C for 30 s, 62 °C for 30 s, and 72 °C for 30 s; 10 cycles at 94 °C for 30 s, 60 °C for 30 s, and 72 °C for 30 s, followed by the final extension at 72 °C for 5 min. The reaction products were separated on a 2% agarose gel.

### Real-time reverse transcription PCR

Real-time RT-PCR was conducted according to the two-step protocol developed by Singh et al. [13]. Total RNA was isolated by short magnetic capture and was reverse transcribed as described above. The real-time PCR was carried out on a CFX96 Touch™ Real-Time PCR Detection System (Bio-Rad Ltd.) using an AmpliQ Real-Time PCR OptiProbe kit or an AmpiQ Real-Time PCR SYBR Green kit (Novazym Polska s. c.). The final reactions (10 µl) contained 2.5 µl of cDNA, 5 µl of AmpiQ Real-Time PCR reagent, 0.15 µl of a 20 µM stock of forward and reverse primers, and 0.15 µl of 10 µM probe in the probe-based assays, or nuclease-free water in the dye-based assays. Different sets of primers were tested, including (i) the PVY-specific primers and TaqMan probe (TM) designed by Singh et al. [13], (ii) the Y4 F3 and B3 primers from the Y4 primer set (TM), combined with the TaqMan probe [13], and (iii) the Y4 F3 and B3 primers from the Y4 primer set in the presence of SYBR Green (SG) (for sequences, see Table 1). The conventional RT-PCR was performed with the Y4 F3 and B3 primers from the Y4 primer set. The thermal cycling steps were 10 min at 95 °C followed by 60 cycles of 10 s at 95 °C and 20 s at 60 °C. Reactions containing only water (no-template control) or cDNA transcribed from total RNA purified from virus-free plants were included in each run as controls. The fluorescence was monitored on the FAM channel and the results were interpreted in terms of Ct (cycle threshold values) determined used default settings in CFX Manager Software (Bio-Rad Ltd.).

### Sensitivity of the RT-LAMP assay

The sensitivity of the RT-LAMP assay was determined by two separate approaches. The first approach was based on the isolation of total RNA by silica capture. The total RNA concentration was adjusted to 100 ng/ml, and tenfold dilutions were prepared down to 1 fg/ml. One microliter of the RNA sample was then amplified by RT-LAMP using the Y4, Y5 and N primer sets. The second approach was based on the serial dilution of the sap extracted from PVY-infected and healthy potato plants. We investigated (i) the impact of the isolation method on the sensitivity of the detection of PVY by the Y4-based RT-LAMP and (ii) the sensitivity of the Y4-based RT-LAMP when compared to real-time RT-PCR. The sap dilutions ranged from tenfold (dilution factor = 1 × 10^1^) to two-million-fold (dilution factor = 2 × 10^6^). For comparison of the sensitivity of the tested methods, the RNA isolation techniques, or the amplification procedures, total RNA was isolated from all prepared sap dilutions by magnetic capture. These RNAs were subsequently tested by RT-LAMP alone or also by real-time RT-PCR according to the procedures described above.

### Comparison of detection methods using field samples

Thirty tubers from 30 offspring plants were harvested after planting secondarily PVY-infected and healthy tubers. The tubers were tested by the Y4-based RT-LAMP method as well as by probe-based and dye-based real-time PCR as described above using the magnetic III protocol for RNA extraction. For the standard grow-out (GO) tests, eye cores were cut, sprouted and grown in a glasshouse to produce 4- to 6 week-old offspring plants. Leaves from these plants were then tested for PVY using the double sandwich enzyme-linked immuno-absorbent assay (DAS-ELISA) according to the manufacturer’s protocol (Neogen).

### Statistical analysis

Each experiment was conducted with at least two replicates and was repeated independently at least three times. A non-template control (sterile water), a negative control (total RNA extracted from a virus-free plant), and a positive control (total RNA extracted from a PVY-infected plant) were included for each LAMP/qPCR run. The time-to-positive (Tp) or Ct values were calculated using the manual baseline settings in Genie II (OptiGene Ltd) or CFX Manager Software (Bio-Rad Laboratories). Mean values and standard deviations were calculated, and the statistical analysis was carried out using GraphPad Prism 6.04 for Windows (GraphPad Software, La Jolla, California, USA, http://www.graphpad.com). An unpaired *t*-test was used to compare the mean annealing temperature (T_a_) values recorded using a Genie II amplification system (OptiGene Ltd) or melting temperature (T_m_) using a CFX96 Touch™ Real-Time PCR Detection System (Bio-Rad Ltd) for PVY isolates representing the N or O coat protein type. Differences with *p* < 0.05 were considered statistically significant.

## Results and discussion

### Performance of the RT-LAMP primer sets

**Fig. 2:**
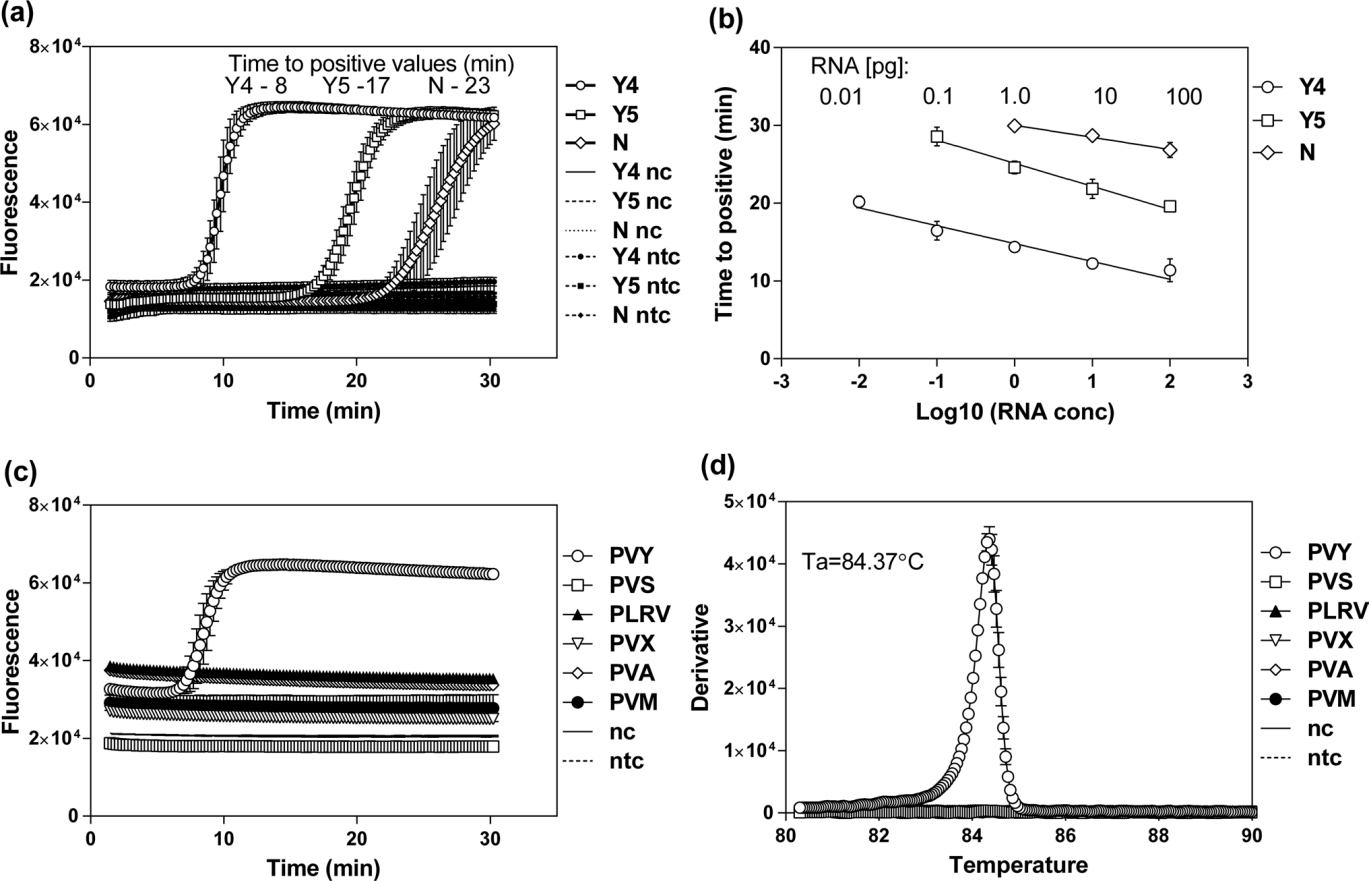
Performance of the Y4-set-primed RT-LAMP for PVY detection. (a) Comparison of the amplification speeds of the Y4, Y5 and N primer sets in the RT LAMP assay for PVY detection from total RNA isolated from plants infected with PVY^NTN^ (isolate 12/94). We used total RNA from virus-free plants (nc) and a no-template control (ntc) as negative controls. The time-to-positive values are shown above the plot. (b) Comparison of the sensitivity of different RT-LAMP assays by serial dilution of total RNA. The RT-LAMP results are shown as time-to-positive (Tp) values as a function of the logarithm of the total RNA concentration. The total RNA (100 pg/µl) was serially diluted down to 0.001 pg/µl. The amounts of RNA [pg] detected by the specific RT-LAMP assays are indicated above the plot. (c) Specificity of the Y4 RT-LAMP for PVY detection expressed as amplification plots for reactions performed using total RNA isolated from plants infected with PVY^NTN^, PVA, PVM, PVS, PVX, or PLRV. (d) Second derivatives of annealing curves recorded for amplicons in the reactions described in panel c. The peak of the plot indicates the annealing temperature (T_a_) of the amplicon produced by Y4 primers for a reaction containing PVY^NTN^ RNA. The data are averages from at least two independent experiments performed in triplicate. Error bars indicate standard deviation

The high specificity of the RT-LAMP method relies heavily on the primer sets designed for the amplification of the targeted region. We thus performed a direct comparison of three RT-LAMP primer sets for PVY detection. These included two primer sets (N and Y5) previously reported in the literature [10, 11] and the newly designed Y4 set (Table 2 and Fig. 1), all of which targeted the coat protein region. While all primer sets successfully amplified the PVY targeted region as expected, their reaction rates were significantly different (Fig. 2a). The slowest amplification was observed for the N set, with a time-to-positive (Tp) value of 23 min. The Y5 primers had a Tp value equal to 17 min. The Y4 set gave the fastest PVY detection with a Tp value of 8 min (Fig. 2a). The specificity of all primer sets was confirmed by the lack of amplification from total RNA isolated from a mock-inoculated plant or a no-template control (Fig. 2a).

To compare the detection sensitivity achieved with the different primer sets, we performed a serial dilution of the total RNA, with the final amount of RNA ranging between 100 pg to 1 fg per reaction. We plotted the time-to-positive values for each primer set as a function of the logarithm of the RNA concentration (Fig. 2b), which showed a linear relationship for all primer sets. The highest sensitivity was obtained with the Y4 set, which successfully detected PVY with as little as 0.01 pg of RNA. Lower sensitivities by one (0.1 pg) and ten orders of magnitude (1 pg) were recorded for the Y5 and N sets, respectively.

The specificity of the Y4 primer set against PVY was confirmed by testing total RNA isolated from leaves infected with other viruses commonly infecting potato, including potato virus A (PVA), potato virus M (PVM), potato virus S (PVS), and potato virus X (PVX), along with PVY. As expected, only PVY was detected (Fig. 2c). No peak was detectable for any other viruses. The annealing temperature of the amplified RT-LAMP product was 84.4 °C (Figure 2d).

We concluded that the newly developed Y4 primer set specifically amplified the targeted region of PVY and that it yielded higher sensitivity than the other tested primer sets, including the previously described Y5 set, which had already been reported to be tenfold more sensitive than the RT-PCR and 1000-fold more sensitive than the enzyme immunoassays [11].

### Discrimination of the PVY O and N types with the Y4 primer set

**Fig. 3:**
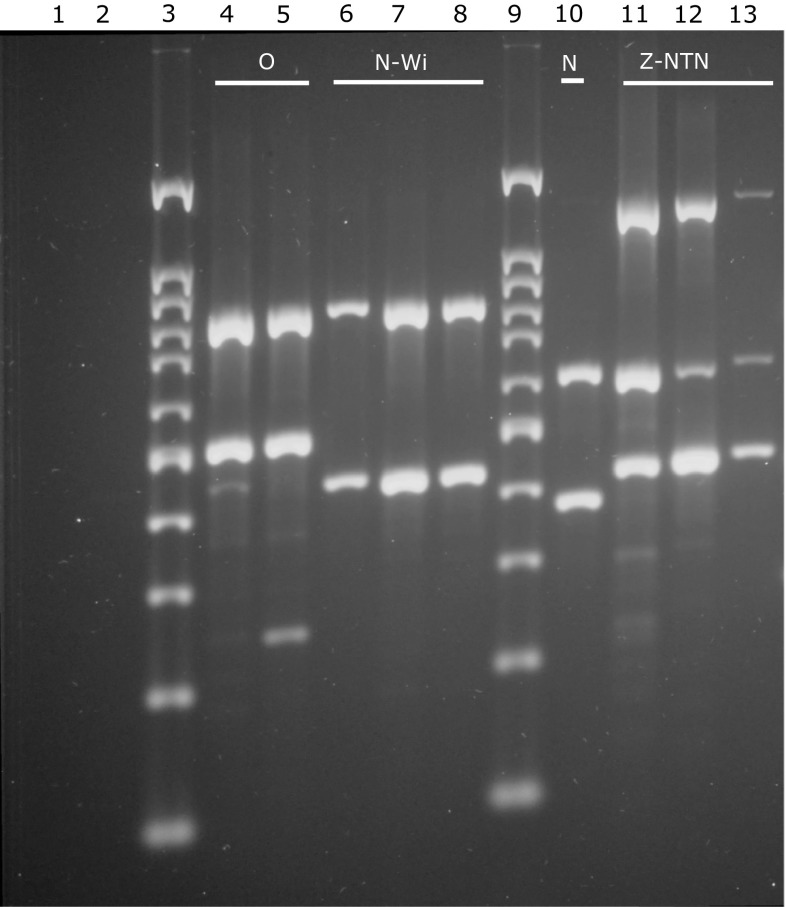
Identification of different PVY strains by multiplex RT-PCR. Lane 1, no-template control, lane 2, RNA from a healthy plant; lane 4, PV-0345; lane 5, Bonin 1; lane 6, Wi (EF558545); lanes 7 and 8, Bonin 2; lane 10, PV-0348 (X97895); lane 11, PV-0403; lanes 12 and 13, Bonin 3; lanes 3 and 9, molecular size markers (Nova 100 bp, Novazym Poland). The isolates PV-0345 and Bonin 1 were identified as PVY^O^, isolates Wi and Bonin 2 as PVY^N-Wi^, isolate PV-0348 as PVY^N^, isolates PV-0403 and Bonin 3 as PVY^NTN^

**Fig. 4:**
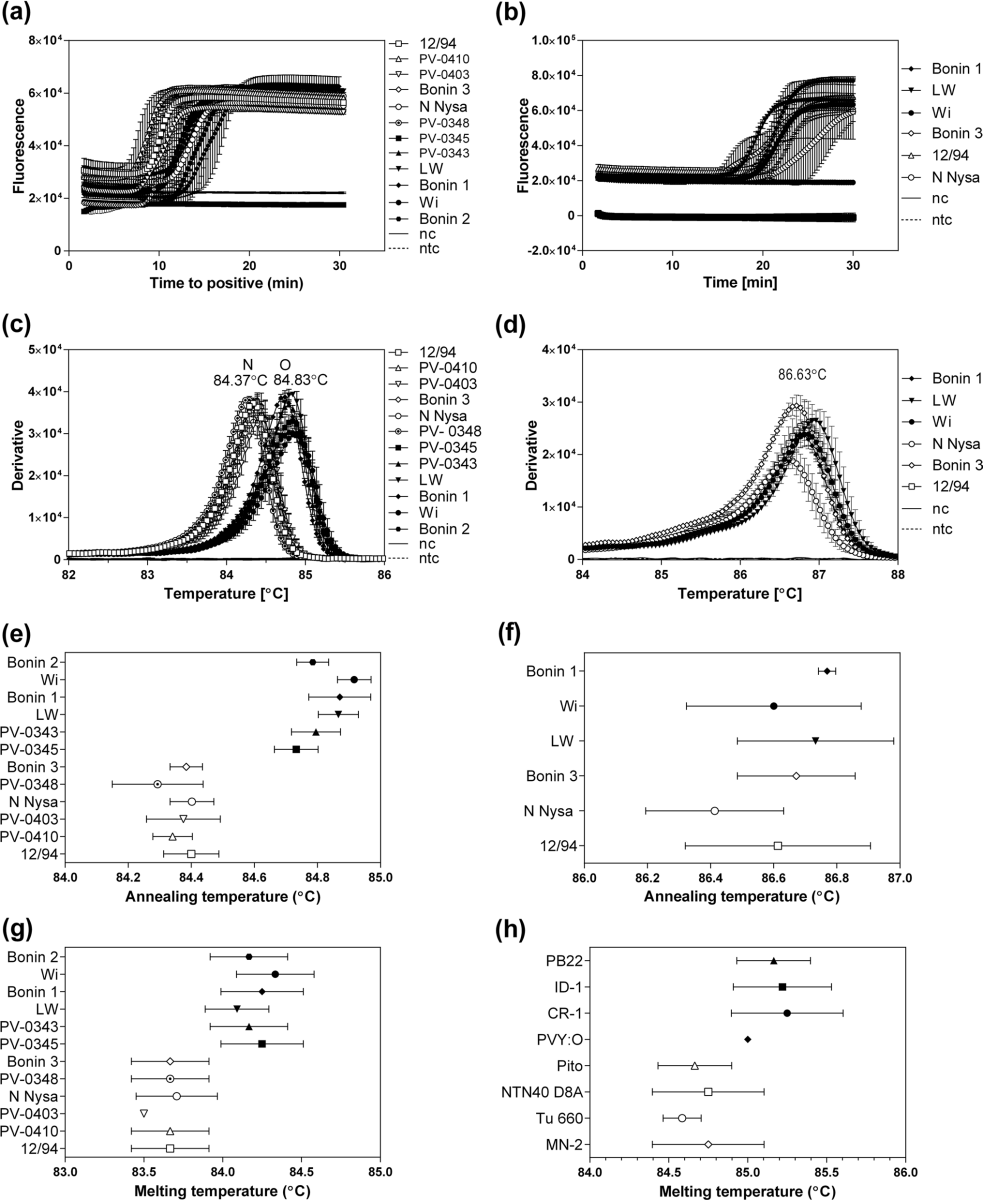
Differentiation of PVY types N and O by the Y4-primer-set-based RT-LAMP. Amplification plots recorded for RT-LAMP reactions with the Y4 (a) and Y5 (b) primer sets and total RNA isolated from plants infected with the indicated European PVY isolates, with total RNA from a virus-free plant (nc), or with water instead of RNA (ntc). Second derivatives of annealing curves recorded for amplicons amplified using the Y4 (c) and Y5 (d) primer sets in reactions described in panels a and b, respectively. Annealing temperature (T_a_) ranges of the RT-LAMP products obtained on European PVY strains by amplification with the Y4 (e) and Y5 (f) primer sets. The annealing temperatures were recorded using a Genie II apparatus (g and h). Melting temperature (T_m_) ranges of the RT-LAMP products obtained using European (g) or North American (h) PVY strains by amplification with the Y4 primer set. The melting temperatures were measured using the Bio-Rad apparatus. PVY strains with coat protein O or N types are indicated by symbols with a black or white background, respectively. The data are averages from at least two independent experiments performed in triplicate. Error bars indicate standard deviation

The ability to discriminate PVY isolates by strain or at least by serotype is a useful tool in disease diagnosis. We thus tested whether the Y4-based RT-LAMP assay could differentiate the PVY isolates that were positive in the multiplex RT-PCR (Fig. 3). These included different PVY^O^, PVY^N-Wi^, PVY^N^ and PVY^NTN^ isolates. We performed the RT-LAMP assay using a Genie II rapid amplification system and the Y4 and the Y5 primer sets (Fig. 4). While no amplification was observed when using total RNA isolated from virus-free plants, strong amplification was detected when using total RNA from all of the PVY-infected plants using both primer sets (Fig. 4a and b). We next assessed strain specificity by comparing the T_a_ values of each of the amplified products (Fig. 4c and e). The resulting curves revealed that the products amplified from PVY isolates with an O serotype, which included PVY^N-Wi^ and PVY^O^, had approximately a 0.46 °C variation in their T_a_ values when compared to the amplified products from the N type isolates, which included PVY^N^ and PVY^NTN^, but this was only observable with the Y4 primer set (*p* < 0.0001, Table 4). The average T_a_ value was about 84.37 °C for the N-type and 84.83 °C for the O-type (Table 3, Fig. 4c). Such significant temperature variation was not observed with the Y5 primer set (difference, 0.14 °C, *p* = 0.2191, Table 3, Fig. 4d and f). The average T_a_ value for all amplified products was 86.63 °C. Thus, the Y5 set detected PVY, but in contrast to the Y4 set, it failed to differentiate the PVY isolates (Table 3, Fig. 4d and f).

**Table 3:** Statistical analysis of differences between mean annealing (T_a_) and melting temperatures (T_m_) recorded for O- and N-type PVY isolates by Y5 and Y4 RT-LAMP assays. T_a_ values were determined using a Genie II rapid amplification system (OptiGene Ltd.), and T_m_ values were determined using a CFX96 Touch™ Real-Time PCR Detection System (Bio-Rad Laboratories, Inc.). EU, European PVY isolates; USA, USA PVY isolates

Unpaired *t*-test data	Y5 RT-LAMP	Y4 RT-LAMP	Y4 RT-LAMP	Y4 RT-LAMP
O vs. N	EU T_a_ ^a^	EU T_a_ ^b^	EU T_m_ ^b^	USA T_m_ ^c^
*P*-value	0.2191	< 0.0001	< 0.0001	0.0005
*P*-value summary	ns	****	****	***
Significantly different (*P* < 0.05)?	No	Yes	Yes	Yes
One- or two-tailed *P*-value?	Two-tailed	Two-tailed	Two-tailed	Two-tailed
*t*, df	*t* = 1,456 df = 4	*t* = 13.56 df = 10	*t* = 12 df = 10	*t* = 6.89 df = 6
How big is the difference?				
Mean ± SEM of N isolates	86.56 ± 0.0786, n = 3	84.37 ± 0.01746, n = 6	83.65 ± 0.03038, n = 6	84.69 ± 0.03961, n = 4
Mean ± SEM of O isolates	86.7 ± 0.05132, n = 3	84.83 ± 0,02937, n = 6	84.21 ± 0.03544, n = 6	85.16 ± 0.05577, n = 4
Difference between means	0.1367 ± 0.09387	0.4633 ± 0.03417	0.56 ± 0.04668	0.4712 ± 0.0684
95% confidence interval	− 0.124 to 0.3973	0.3872 to 0.5395	0.456 to 0.664	0.3039 to 0.6386
R squared (eta squared)	0.3464	0.9484	0.935	0.8878
F test to compare variances				
F, DFn, Dfd	2.346, 2, 2	2.829, 5, 5	1.361, 5, 5	1.982, 3, 3
*P*-value	0.5977	0.2784	0.7433	0.5883
*P*-value summary	ns	ns	ns	ns
Significantly different (*P* < 0.05)?	No	No	No	No

^a^ The mean O group T_a_ value was calculated from mean T_a_ values recorded for O isolates (Bonin 1, Wi, LW) and mean N group T_a_ was calculated from mean T_a_ values recorded for N isolates (Bonin 3, N Nysa, 12/94)

^b^ The mean O group T_a_ or T_m_ value was calculated from T_a_ or T_m_ values recorded for O isolates (Bonin 2, Wi, Bonin 1, LW, PV-0343, PV-0345), and the mean N group T_a_ or T_m_ value was calculated from T_a_ or T_m_ values recorded for N isolates (Bonin 3, PV-0348, N Nysa, PV-0403, PV-0410, 12/94)

^c^ The mean O group T_m_ was calculated from mean T_m_ values recorded for O type isolates (PB22, ID-1, CR-1, PVY:O), and the mean N group T_m_ value was calculated from T_m_ values recorded for N isolates (Pito, NTN40 D8A, Tu 660, MN-2)

To determine whether the observed difference in T_a_ values with the Y4 primers (Fig. 4c and e) correlated with a difference in melting temperature (T_m_), all PVY strains were tested using a real-time thermal cycler (Fig. 4g). Our data revealed that the PVY strains with an N-type coat protein sequence had a T_m_ value of 83.65 °C, while strains with an O-type coat protein sequence had a T_m_ value of 84.21 °C (difference, 0.56 °C, *p* < 0.0001, Table 3, Fig. 4g).

We expanded the RT-LAMP assays with the Y4 primers to include North American PVY^O^, PVYN^N:O^, PVY^N-NA^ and PVY^NTN^ isolates (Fig. 4h). The melting curves revealed that the products of the RT-LAMP amplification obtained for PVY^O^ and PVYN^N:O^ with the O-type coat protein gene had a T_a_ value (85.16 °C) that was 0.47 °C higher than that of the products obtained for PVY^N-NA^ and PVY^NTN^ (84.69 °C) (*p* = 0.0005, Table 3, Fig. 4h).

In summary, the Y4-based RT-LAMP set allowed us to discriminate PVY O and PVY N types in one reaction. Sequence analysis (Fig. 1) showed that the amplified region within the coat protein gene contains two single and two double nucleotide signatures typical for O and N coat protein types, which may be sufficient to contribute in the differences in annealing/melting temperatures that we observed between the N and O types (Table 3, Fig. 4).

### Optimization of the RNA isolation procedure

**Fig. 5:**
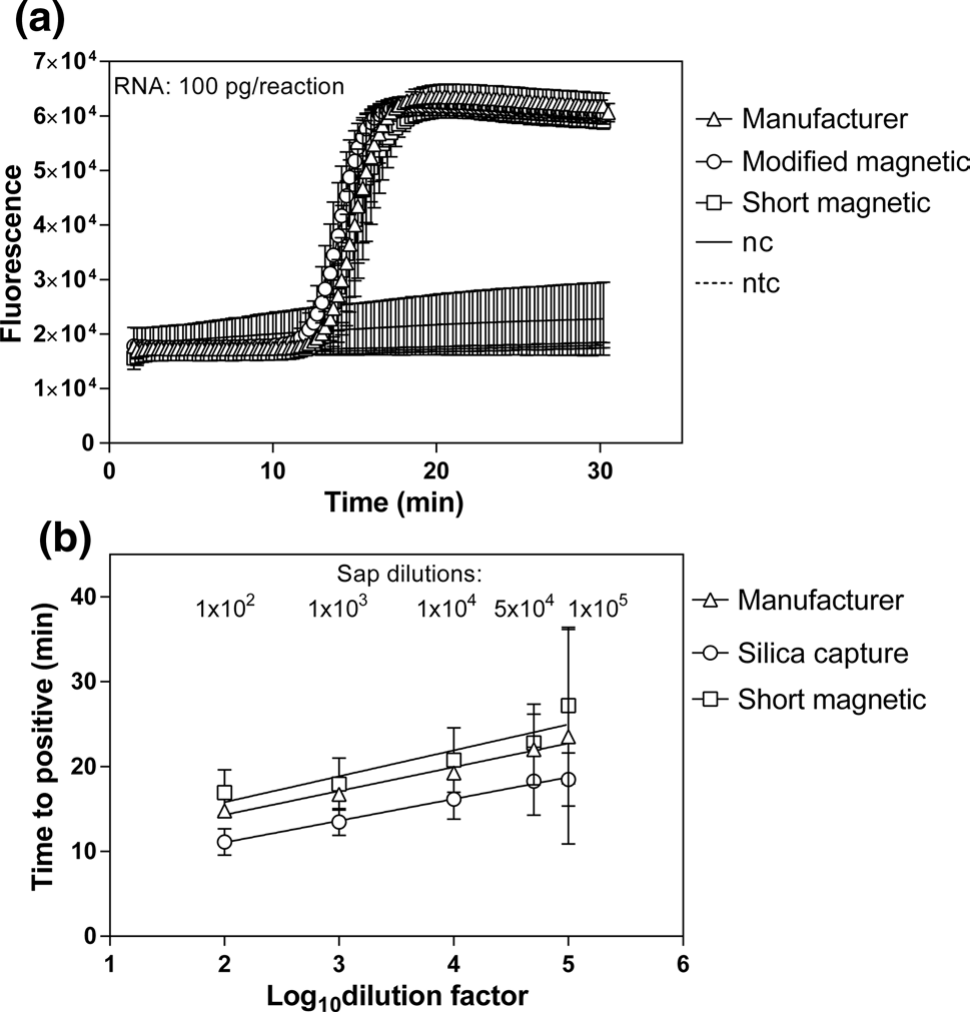
Effect of the method of RNA isolation on the performance (a) and sensitivity (b) of detection of PVY by Y4 RT-LAMP. (a) Amplification plots recorded for RNA purified from plants infected with PVY by magnetic capture (i) according to the manufacturer (ii) by a modified magnetic procedure and (iii) by a shortened magnetic procedure. (b) Comparison of the effect of the different RNA isolation methods on sensitivity. Sap from plants infected with PVY was serially diluted up to 2 × 10^6^ times, and total RNA was purified from each dilution by the magnetic capture procedure recommended by the manufacturer, by the shortened magnetic capture procedure, and by silica capture. Time-to-positive results are shown as a function of the logarithm of the dilution factor. The data are averages from at least two independent experiments performed in triplicate. Error bars indicate standard deviation

Our next step was to streamline the RT-LAMP procedure. We thus compared different RNA isolation procedures, including magnetic capture and RNA silica capture, that we previously optimized for RT-PCR [13]. We first compared three versions of the magnetic-capture-based RNA isolation procedure as described in Materials and methods. These included (i) the magnetic capture following the manufacturer’s procedure, (ii) a modified magnetic capture with buffers adapted for silica capture [13], and (iii) a shortened modified magnetic capture with fewer washes and incubation steps than the modified capture procedure. We observed that all these procedures resulted in nearly identical amplification profiles in RT-LAMP (Fig. 5a). However, we shortened the procedure from start to completion from 30 and 60 min with the original and modified method, respectively, to 20 min with the shortened isolation approach.

To test whether the technique of RNA isolation would influence the sensitivity of PVY detection by RT-LAMP, we compared the original magnetic capture method and the shorter magnetic capture method with the silica capture method, which resulted in high sensitivity in the RT-PCR assay [13] (Fig. 5b). Our data showed that all isolation methods had the same detection limit (a 1 × 10^5^-fold dilution of sap), but the time-to-positive values were slightly higher with RNA isolated by magnetic capture than with RNA isolated by the silica-based RNA capture procedure (Fig. 5b).

In summary, we reduced the time required for the magnetic capture procedure for PVY detection by RT-LAMP from RNA isolation to assay to less than one hour without compromising the detection sensitivity.

### Comparison of the sensitivity of PVY detection by RT-LAMP and by conventional and real-time RT-PCR

**Fig. 6:**
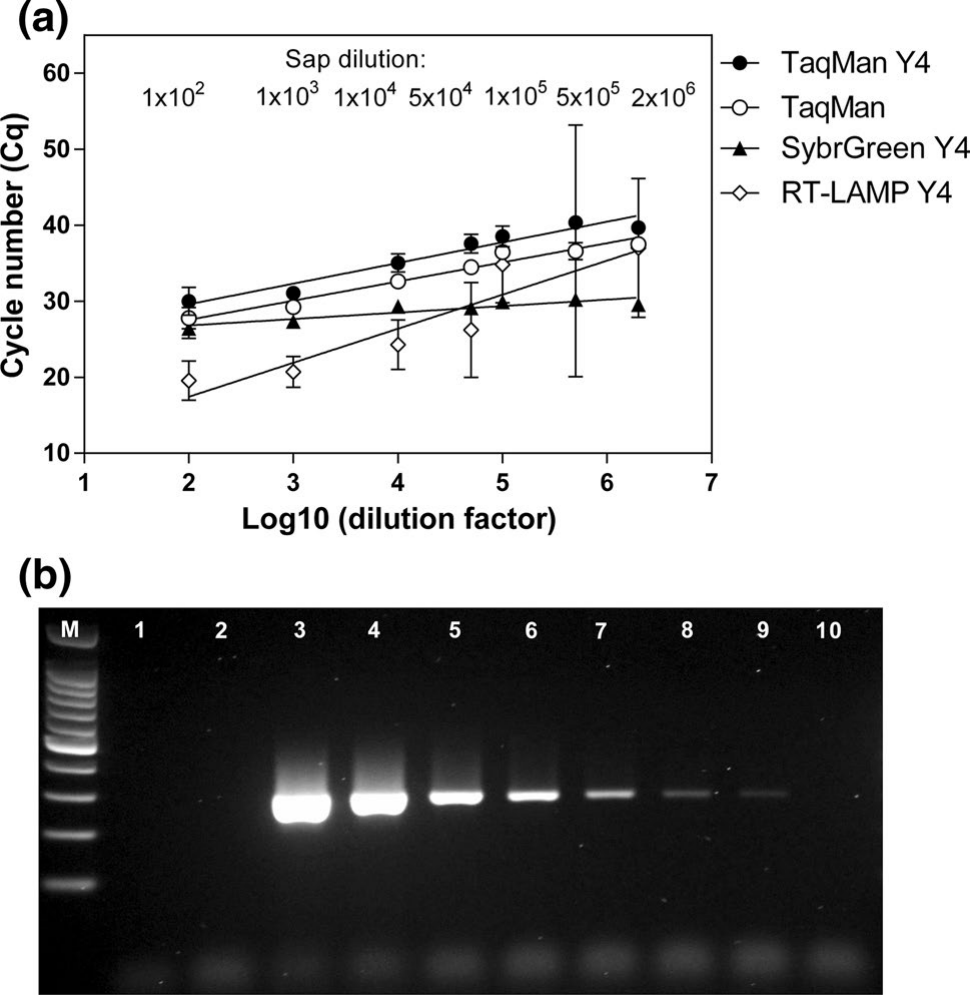
Comparison of sensitivity of the Y4 RT-LAMP, the TaqMan real-time RT-PCR, the Y4 TaqMan real-time RT-PCR, the Y4 SYBR Green real-time PCR (a), and the conventional Y4 RT-PCR (b). (a) For the real-time tests, Cq results are shown as a function of the logarithm of the dilution factor. The data are averages from at least two independent experiments performed in triplicate, and error bars indicate standard deviation. (b) Determination of the sensitivity of PVY detection by RT-PCR in sap from a PVY-infected plant diluted 1 × 10^2^, lane 3; 1 × 10^3^, lane 4; 1 × 10^4^, lane 5; 5 × 10^4^, lane 6; 1 × 10^5^, lane 7; 5 × 10^5^, lane 8; 1 × 10^6^, lane 9; 2 × 10^6^, lane 10. No-template control (PCR reaction containing water instead of cDNA), lane 1; negative control (RT-PCR reaction containing RNA from a virus-free plant), lane 2; Nova 100-bp molecular weight marker (Novazym Poland), lanes M

Next, we compared the sensitivity of the Y4-based RT-LAMP for PVY detection with that of real-time RT-PCR and conventional RT-PCR using serial dilutions of sap. The data revealed that the RT-LAMP was as sensitive as all investigated variants of the real-time RT-PCR, with the detection of PVY in sap diluted up to 2 × 10^6^ fold in fewer than 40 cycles (Fig. 6a). However, the detection of PVY at the highest RNA concentration (1 × 10^2^-fold dilution) required more than 25 cycles for all tested real-time RT-PCR variants but only 19 cycles by RT-LAMP. To contrast, the conventional RT-PCR amplified a PCR product of the expected size in samples diluted only up to 1 × 10^6^-fold (Fig. 6b).

While the RT-LAMP assay shares similar sensitivity with the standard real-time RT-PCR assay, it is clearly faster. The detection of PVY in the highest dilution of sap can be accomplished in 15-60 min by RT-LAMP with its 30-s cycles. In contrast, the completion of the real-time RT-PCR reaction with the same sample requires 2-3 h.

### Reliability of PVY detection in dormant tubers

To estimate the reliability of the RT-LAMP for detection of PVY in dormant tubers, tubers collected from secondarily infected plants were tested for the presence of PVY by RT-LAMP (Y4), by the two variants of real-time RT-PCR (dye-based [SG] and probe-based [TM]) and by the standard grow-out test (GO). While all 30 tubers tested positive for PVY by the GO test, 26 tubers tested positive by RT-LAMP while only 19 tubers tested positive by both variants of real-time RT-PCR (Table 4). Our data suggest that while the grow-out test remains a reliable method for the detection of PVY in dormant tubers, the RT-LAMP assay clearly out-performed the real-time RT-PCR assay. This may be linked to the requirement for high-quality RNA preparations for PCR-based amplification, which can be a challenge with the low virus titer in dormant tubers [3]. It is worth noting that some differences were observed between the real-time PCR variants depending on whether the detection was dye- or probe-based (Table 4).

**Table 4:** Comparison of different detection methods for testing of field samples

No.	Grow-out	Direct tuber testing		
	DAS-ELISA	RT-LAMP	SG Real-Time RT-PCR	TM Real-Time RT-PCR
1	**+**	**−**	**−**	**−**
2	**+**	**−**	**+**	**−**
3	**+**	**+**	**+**	**+**
4	**+**	**+**	**−**	**+**
5	**+**	**−**	**−**	**−**
6	**+**	**+**	**+**	**+**
7	**+**	**+**	**−**	**+**
8	**+**	**+**	**+**	**+**
9	**+**	**+**	**+**	**+**
10	**+**	**+**	**−**	**−**
11	**+**	**+**	**−**	**+**
12	**+**	**+**	**+**	**+**
13	**+**	**+**	**+**	**+**
14	**+**	**+**	**+**	**+**
15	**+**	**−**	**−**	**−**
16	**+**	**+**	**+**	**+**
17	**+**	**+**	**+**	**+**
18	**+**	**+**	**+**	**+**
19	**+**	**+**	**+**	**+**
20	**+**	**+**	**+**	**+**
21	**+**	**+**	**+**	**+**
22	**+**	**+**	**+**	**+**
23	**+**	**+**	**+**	**+**
24	**+**	**+**	**+**	**+**
25	**+**	**+**	**−**	**−**
26	**+**	**+**	**−**	**−**
27	**+**	**+**	**+**	**−**
28	**+**	**+**	**−**	**−**
29	**+**	**+**	**−**	**−**
30	**+**	**+**	**+**	**−**
Ratio	30/30	26/30	19/30	19/30
